# Modern Views on Heredity

**Published:** 1909-05-01

**Authors:** R. Murray Leslie

**Affiliations:** Senior Physician to the Prince of Wales's General Hospital, Physician to the Royal Hospital for Diseases of the Chest


### Hospital Clinics.

### MODERN VIEWS ON HEREDITY.

By R. MURRAY LESLIE, M.A., B.Sc., M.D., M.R.C.P.; Senior Physician to the Prince of Wales's General Hospital, Physician to the Royal Hospital for Diseases of the Chest.

(A Lecture Delivered at the N.E. London Post-Graduate College.)

(*Concluded from page* 61.)

### Immunity from Microbic Diseases.

As Sir William Church well expresses it, "no disease which arises from or is associated with the presence of a foreign body, living or dead, within us can be considered hereditary." Thus *syphilis* is not a hereditary disease. Congenital it certainly may be, as the contagion can pass from mother to child. This is not heredity, but intra-uterine contagion. It is undoubtedly true that in certain families and races there is a tendency to, or an immunity against, various kinds of bacterial disease. Certain families seem to afford a more favourable home for the invading organism. Immunity from such disease appears to be associated with certain bio-chemical changes in the body fluids. Does a race acquire and transmit an immunity to a specific microbial disease? How else can we explain the extinction of *leprosy* in England, which was formerly endemic throughout the country?

*Influenza* is a much less fatal disease at the present time than it was when it was reintroduced into England in 1890. *Measles*—a comparatively mild disease in Europe—manifests great malignity when introduced into virgin soil amongst savage tribes where the disease has been previously unknown. Instances might be multiplied indefinitely in support of the view that immunity can be inherited. Ehrlich expressed the interesting opinion that the relative degree of immunity to certain bacterial diseases may in part be due to the infant imbibing in its mother's milk certain protective substances derived from the body cells. Whether this be true or not, there is abundant evidence in support of the generally accepted belief in the doctrine of a gradual acquirement by inheritance of an increase of racial immunity.

*Tuberculosis* as a disease is never hereditary. The organs of new-born children are sterile. Although a few rare cases have been described of children born tubercular, it does not in any sense follow that the disease was inherited. It simply means that the mother may have suffered from genital tuberculosis, and as a result there was accidental transmission of the tubercle bacillus to the child *in utero*. It has been asserted that the ovum at the time of impregnation may become infected through the spermatic fluid emanating from a man suffering from tuberculosis of the testicle. No proof of such an occurrence has been adduced.

The question of the hereditary transmission of a predisposition to tuberculosis stands on quite a different footing. Even here, however, many recent authorities have gone so far as to disclaim all belief in hereditary predisposition. They allege that when a member of a household becomes tubercular the tendency of infection of other members through frequent association is necessarily much greater, and that this constant exposure to infection sufficiently accounts for the disease selecting particular families and, it may be, affecting several generations in succession.

A review of the general evidence, taken in conjunction with my own experience as a physician to a consumption hospital, makes me entirely dissent from this view. I am convinced that opportunity of infection, though by far the most important factor, is not the only one to be considered in the incidence of tuberculosis. It is a matter of common knowledge that the disease attacks unequally those who are equally exposed to the same external conditions of infection, and it is not necessarily persons debilitated through malnutrition or from some other cause who are attacked. Members of certain families are particularly susceptible and are prone to be attacked, even if placed in favourable conditions.

The results of hospital treatment are certainly more unfavourable among patients with a strong tubercular family history. Though I am convinced that there is this hereditary proclivity to the disease, yet it is so much less important a factor than opportunity of infection that in my opinion there is no valid reason for anyone to abstain from marriage simply because there is a marked family history of consumption. I would go even further and allege that there is no grave objection to a young man marrying who himself exhibits evidence of arrested disease. It is different in the case of a young woman, who with latent disease is apt to have an acute exacerbation after childbirth. Even in the latter case, the child will be born perfectly healthy, and if placed in suitable hygienic conditions will probably never develop the disease.

What actually is inherited is most probably a vulnerability of the protective epithelia, and accordingly a diminished resistance to the inroads of the ubiquitous bacillus. It is possible that the diminishing incidence of the disease may be due in some measure to a partial immunity connected with bio-chemical changes in our bodies and inherited in the course of generations from tuberculous ancestors in whom the disease has been cured. Professor Calmette was certainly successful in producing such immunity in certain animals.

### Gout and Heredity.

It is commonly believed that this disease is engendered by high living and that when once established it is capable of being transmitted hereditarily. Probably what is inherited is a special variation which expresses itself in an altered mode of eliminating nitrogenous waste-products. This tendency is simply rendered manifest by certain external agencies such as dietetic excesses. Dr. Garrod has in his recent Croonian lectures drawn attention to the complex nature of what he terms "inborn errors of metabolism," and brings forward evidence indicating that certain errors in metabolism such as cystinuria and alkaptonuria appear to run in families, and suggests that further research may determine how far their manifestations may be in accordance with Mendel's law of heredity. The gouty habit of body may have arisen as a variation, and as such is transmissible by inheritance.

### Cancer.

Cancer is still popularly believed to be a typical hereditary disease. There is no evidence whatever that the actual disease can be transmitted by inheritance, and no very definite proof even of hereditary predisposition. It is known that certain species among the mammalia are much more liable to certain forms of cancer than others. Thus cancer of the breast, so common in women, dogs, and mice, is practically unknown in the cow, which, however, frequently suffers from cancer of the liver. Such tendencies would seem to depend upon innate characters transmissible by inheritance. The examination of statistics in connection with cancer in man has induced such an authority as Weinberg to conclude that heredity does not play a prominent part in the incidence of cancer. Dr. Bashford states that there is no evidence of cancer arising as a transmissible variation, and believes that the disease is probably always acquired. Experimental evidence in the case of cancer in mice strongly supports Dr. Bashford's contention. Mr. Butlin, on the other hand, thinks that some individuals have a special inborn susceptibility to cancer.

### Nervous and Mental Diseases.

It is an undeniable fact that many forms of nervous disease are markedly hereditary. This is admitted by practically all alienists and neurologists. Sir William Gowers recently asserted that in at least 50 per cent. of cases of *epilepsy* and *insanity* there is a family history of one or both maladies, in antecedents and present or past collaterals.

Rohde, in 1895, argued that in all such cases a special germinal variation is inherited which may express itself in various ways—*e.g.* in a general neurasthenia or in an acute neurosis such as mania, in the presence of a sufficiently powerful exciting cause. Thus the functional disturbance associated with puberty may predetermine an attack of acute hysteria and epilepsy, while the stress of early adolescence superadded to profound emotion may induce an attack of acute mania.

A neurotic heredity—according to Clouston—consists in the inheritance of general morbid tendencies rather than direct proclivities to special diseases. As proving the fact of an hereditary psychopathic constitution, he refers to the family history of one of the European Royal families which for 200 years has exhibited manifestations of this constitution in nearly all its varieties.

As regards the hereditariness of neuroses, Weismann himself makes the important admission that environment, including dietetic conditions, may affect the germ-plasm. If nutritive disturbances can affect the germ-plasm it is only reasonable to suppose that the embryo may be affected disadvantageously through the mother. Clouston considers that a good heredity consists in the power of the fertilised ovum to integrate, develop, and maturate along normal lines from the first fusion of the germ and sperm cells up to the full development of the individual. If this power be debased by altered nutrition or in any other way, there is at once a practical explanation of many neuroses. According to this observer the most important neuroses arise during the period of development owing to some defect in the maturation of the cells and tissues. A consideration of the marvellous structure and functions of a typical brain cell (of which there are about 400,000,000 in each individual) makes it apparent that a very small deficiency in the nutrition of the germ-plasm may readily result in the arrest or debasement of the developmental process in groups of these cells. Hereditary neuroses, according to Clouston, are due to arrests of maturation of the cells that constitute the nerve centres. Certain individuals exhibit weakness in the nutrition of the germ cells, and accordingly have not the power to develop into full maturity, this defect recurring in successive generations.

It is a fact that a large proportion of the cases of epilepsy and other neuroses occur during the period of development, while insanity in its most marked and characteristic phases also occurs during the developmental period. The strain associated with the sexual and reproductive instinct at adolescence proves too severe for the defective power of resistance of non-matured or debased brain-cells. It is this tendency to arrested or imperfect maturation of brain-cells that is handed down to successive generations. It is rarely that a specific form of insanity is transmitted: it is more often a general nervous instability.

As regards *dipsomania*, there is no doubt of the fact, whatever may be the explanation, that in certain families there is a tendency to alcoholic excess. Dr. Clouston holds that no man is born with a craving for alcohol, but individuals are born who exhibit a morbid reactiveness to alcoholic stimulants and a lack of the higher inhibitory mental powers. A man with such a neurotic or psychopathic tendency would, by indulging in even a small quantity of alcohol, lose mental power and strength of mind with each repetition of the dose, and in time would degenerate into a confirmed dipsomaniac. It is a special nervous instability and not a love of alcohol which is inherited.

Certain special forms of nerve diseases—more particularly the myopathies—are markedly hereditary. One might specially mention *hereditary ataxy* (*Friedreich's disease*), *Marie's ataxia*, *Déjerine's facial paralysis*, *Thomsen's disease*, *Huntingdon's chorea*, *congenital optic atrophy*, and *night-blindness*. Some of these diseases have been traced through as many as five generations, and the incidence is sometimes strikingly in accordance with Mendel's "Law of heredity."

### Sex Limited Diseases.

We shall now refer to the interesting group of diseases manifesting a maternal heredity. It includes *pseudo-hypertrophic paralysis*, *colour blind-*

*ness*, *hæmophilia*, and *hereditary optic atrophy*. In the case of pseudo-hypertrophic paralysis, for instance, the males alone exhibit the disease in a typical form; the heredity is maternal only, the affection never being transmitted by the father. The tendency must therefore be germinal—a potential defect in that part of the ovum which will become the muscles of the male. The female children escape, but they inherit the tendency—or some of them do—and may transmit the disease to their sons. In the ova from which the daughters develop, the potentiality of the disease is not in the elements that develop into muscles, but only in that portion of the protoplasm which will become their germinal tissue and form the muscles of the male offspring.

Colour blindness is found to be a "dominant" character in males, and "recessive" in females. A female will not be colour blind, as Professor Bateson points out, unless she has had two doses of colour blindness, one from each of her parents. The colour-blind female must have had a colour-blind father and all her sons will be colour blind. The male who is not colour blind cannot pass it on, but the male who is colour blind can pass it on, and on an average half his sons will be colour blind and half his daughters will be able to transmit the condition.

*Diabetes insipidus*, *tylosis palmaris*, *polydactylism*, *hay fever*, *congenital asthma*, *congenital cataract*, *baldness*, and *albinism* are also hereditary. Mr. Mudge, at the recent discussion on heredity at the Royal Society of Medicine quotes Dr. Drinkwater's case of congenital asthma, which is quite Mendelian in that the affected persons transmitted the disease, while the unaffected did not, also in that some individuals were asthmatic while others were quite normal. The proportion of asthmatic and normal individuals in the descendants of parents, one of whom in each pair is asthmatic, and the other normal, is that predicted by Mendelian principles. An equal number of each is expected, and if we assume that the original pair consisted of a normal and an abnormal member, then there are ten asthmatic and ten normal descendants.

There is still a great controversy waging as to the application of Mendelism to the incidence of hereditary disease. While Professor Bateson on the one hand is enthusiastic as to its value, Professor Pearson states emphatically that there is no definite evidence of the applicability to man of Mendel's law of inheritance. The matter must for the present remain undecided until there are more collected data on which to form an opinion. In the meantime, the medical profession will do well to consider Professor Pearson's advice, to go on collecting reliable pedigrees, which necessitates a great deal of care and trouble. Dr. Bullock goes so far as to assert that very few pedigrees published during the last 200 years are of any use—certainly not more than 20 from all the hospitals—and adds that when a large number of really good pedigrees are obtained it will be time enough to speak about theories of inheritance.

